# Exploring the Role of Skeletal Muscle in Insulin Resistance: Lessons from Cultured Cells to Animal Models

**DOI:** 10.3390/ijms22179327

**Published:** 2021-08-28

**Authors:** Alessandra Feraco, Stefania Gorini, Andrea Armani, Elisabetta Camajani, Manfredi Rizzo, Massimiliano Caprio

**Affiliations:** 1 Laboratory of Cardiovascular Endocrinology, IRCCS San Raffaele Roma, 00166 Rome, Italy; alessandra.feraco@sanraffaele.it (A.F.); stefania.gorini@sanraffaele.it (S.G.); andrea.armani@sanraffaele.it (A.A.); 2 Department of Human Sciences and Promotion of the Quality of Life, San Raffaele Roma Open University, 00166 Rome, Italy; elisabetta.camajani@uniroma1.it; 3 PhD Programme in Endocrinological Sciences, Department of Experimental Medicine, University of Rome “La Sapienza”, 00161 Rome, Italy; 4 Promise Department, School of Medicine, University of Palermo, 90127 Palermo, Italy; manfredi.rizzo@unipa.it

**Keywords:** myofibers, adipose tissue, glucose metabolism, free fatty acids, glycemia

## Abstract

Skeletal muscle is essential to maintain vital functions such as movement, breathing, and thermogenesis, and it is now recognized as an endocrine organ. Muscles release factors named myokines, which can regulate several physiological processes. Moreover, skeletal muscle is particularly important in maintaining body homeostasis, since it is responsible for more than 75% of all insulin-mediated glucose disposal. Alterations of skeletal muscle differentiation and function, with subsequent dysfunctional expression and secretion of myokines, play a key role in the pathogenesis of obesity, type 2 diabetes, and other metabolic diseases, finally leading to cardiometabolic complications. Hence, a deeper understanding of the molecular mechanisms regulating skeletal muscle function related to energy metabolism is critical for novel strategies to treat and prevent insulin resistance and its cardiometabolic complications. This review will be focused on both cellular and animal models currently available for exploring skeletal muscle metabolism and endocrine function.

## 1. Introduction

Skeletal muscle is one of the most fascinating mammalian organs. Muscles represent about half of total body weight and are essential to maintain vital functions such as movement, postural support, breathing and thermogenesis [1,2]. In addition, skeletal muscle represents one of the most dynamic and plastic tissues in humans. Mammalian skeletal muscle is mostly composed of long, postmitotic, multinucleated myofibers, whose selective functional recruitment allows muscles to accomplish their functional duties. Interestingly, myofibers are able to perform adaptive changes in terms of structure and function, thanks to endogenous sensors that act as detection system [3]. Such ability is essential to properly respond to stimuli arising from neural stimulation, energy substrates, and hormonal signals [4]. Notably, muscles are primary sites for glucose uptake and storage, and they also represent a reservoir of amino acids in the form of proteins. Skeletal muscle has a major contribution to whole-body energy metabolism, since it is a crucial consumer of glucose; therefore, its metabolic derangements play a pivotal role in the development of insulin resistance and type 2 diabetes (T2D) [5].

Due to skeletal muscle properties, the establishment of adequate models to investigate molecular signaling pathways, regulating muscle differentiation and metabolic/endocrine function, might allow identifying novel targets for pharmacological therapies against skeletal muscle metabolic dysfunctions. This review aims to summarize the most important in vitro and in vivo models developed to investigate the role of skeletal muscle in glucose homeostasis and insulin resistance, focusing on the molecular mechanisms related to muscle differentiation and endocrine function.

## 2. Skeletal Muscle: A Veritable Endocrine Organ

**Figure 1:**
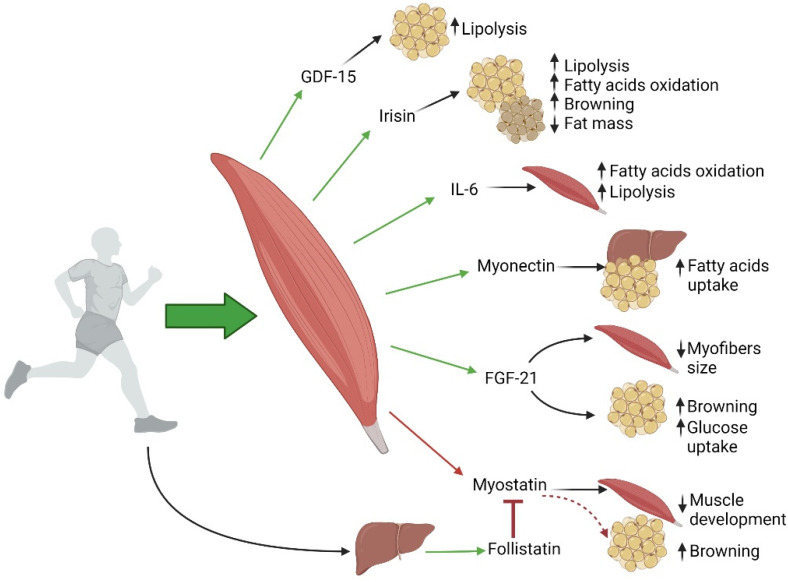
Skeletal muscle as an endocrine organ: paracrine and autocrine effects of muscle contraction-induced myokines. Exercise stimulates the release of myokines with autocrine, paracrine, or endocrine functions (green arrows, induction). In particular, several myokines play a role in the maintenance of whole-body metabolic homeostasis. GDF-15, IL-6, and irisin stimulate intramyocellular triacylglycerol lipolysis. Irisin and FGF-21 induce browning in adipose tissue. Irisin and IL-6 also stimulate fatty acid oxidation. Myonectin affects adipose tissue and liver functions, increasing fatty acids uptake. Myostatin is a negative regulator of skeletal muscle mass and inhibits muscle hypertrophy: exercise induces a downregulation of myostatin (red arrow), thus promoting muscle development. In addition, follistatin, expressed by the liver in response to physical activity (green arrow, induction), is able to antagonize myostatin (red line, inhibition), promoting browning of WAT (dotted red arrow). Black arrows indicate positive (up) or negative (down) modulation of downstream targets. Abbreviations: FGF21, fibroblast growth factor 21; IL-6, interleukin-6; GDF-15, growth differentiation factor 15.

Skeletal muscle secretes a variety of molecules (cytokines and peptides), denominated “myokines”, which act in an autocrine, paracrine, or endocrine hormone-like manner [6]. For this reason, skeletal muscle is now recognized as an endocrine organ. In particular, such active secretion plays a pivotal role in the cross-talk between skeletal muscle and different organs, including white and brown adipose tissue, liver, pancreas, heart, vessels, bones, and brain. Notably, several myokines are synthesized and released by myocytes in response to muscular contractions [7]. More specifically, acute and chronic physical activity result in different modulations of myokine expression and secretion [8]. Indeed, the release of myokines mediates the healthy effects of physical exercise, whereas physical inactivity seems to impair myokines secretion and represents a potential mechanism to explain the correlation between sedentary lifestyle and many chronic diseases [9]. On the other hand, altered secretion of several myokines has been shown to be a hallmark of skeletal muscle in T2D, suggesting a potential role of myokines in the response of skeletal muscle to impaired insulin sensitivity and mitochondrial dysfunction [10]. Myokines are implicated in the autocrine regulation of muscle metabolism as well as in the paracrine regulation of other organs expressing myokines receptors, such as brain, liver, and adipose tissue [11] (Figure 1). Myostatin (MSTN) was the first myokine identified in 1997 [12]. Human skeletal muscle is known to secrete more than 600 myokines [13]. Of note, although a large number of myokines has been described in proteomic studies, we currently have a rather limited knowledge of their biological activity and function. Among the most studied myokines, MSTN, also known as growth differentiation factor 8, is a member of the transforming growth factor-beta super-family and is expressed in the developing and adult skeletal muscle, acting as a negative regulator of muscle development [14]. Mice overexpressing *MSTN* in skeletal muscle show decreased muscle mass with a parallel increase in adipose tissue mass [15]. Conversely, MSTN-null mice show muscle hypertrophy as well as hyperplasia associated to reduced intramuscular adipose tissue compared to controls. Interestingly, such phenotype has been correlated with increased energy expenditure, despite reduced leptin circulating levels in these mice [12,16,17]. Accordingly, *MSTN* deletion resulted in enhanced peripheral tissue fatty acid oxidation and increased thermogenesis, also promoting brown adipose tissue (BAT) activation in mice fed a high-fat diet [18]. Nevertheless, the impact of MSTN is still controversial, with evidence that this myokine may trigger different effects on adipogenesis in vivo and in vitro [16,19]. However, it has been shown that pharmacological administration of MSTN does not reduce adipose mass in adult mice [20], whereas another study demonstrated that recombinant MSTN promotes the differentiation of multipotent mesenchymal cells into the adipogenic lineage, thereby inhibiting myogenesis [21]. In particular, it has been demonstrated that MSTN can mimic glucocorticoid proadipogenic effect in vitro if added to the culture medium of mesenchymal cells at very early stages of adipogenic differentiation, although MSTN-induced white adipocytes appeared smaller compared to those obtained under classical differentiation protocol. Accordingly, mice overexpressing *MSTN* under the control of the aP2 promoter (which is active in mesenchymal cells of mouse bone marrow) show reduced white adipocyte size and improved glucose tolerance as well as insulin sensitivity [22]. On the other hand, several studies indicate that MSTN inhibition can induce browning in white adipose tissue (WAT), i.e., the process of formation of brown adipocyte in WAT depots [23]. Indeed, *MSTN* deletion has been shown to induce BAT-specific protein and gene expression in adipose tissue and skeletal muscle in vivo [24].

Follistatin (FST) is expressed by the liver in response to physical activity and is known to antagonize MSTN and to promote browning, inducing the expression of thermogenic markers and increasing respiratory function both in vitro and in vivo [25,26]. Interestingly, it has been shown that MSTN inhibition induces the browning of WAT through activating the AMPK–PGC1α–FNDC5 pathway in skeletal muscle [27]. Irisin, identified in 2012 by Spiegelman’s group as a new myokine driving thermogenesis and the browning of white fat, is the result of the proteolytic cleavage of the FNDC5 protein [28]. It is secreted by skeletal muscle in response to high-intensity or resistance exercise [29], thus mediating some beneficial effects of physical training in humans, including weight loss, thermoregulation, and improved glucose tolerance. In addition to physical exercise, diet and hormones regulate irisin secretion [30]. To date, no specific receptor for irisin has been identified, but in some tissues, such as bone and fat, irisin exerts its action through integrins, which are widely expressed transmembrane receptors [31]. In addition to its capability to induce browning in adipose tissue, with subsequent favourable metabolic consequences, irisin also plays an important role in the central nervous system exerting anti-inflammatory effects and protecting from neuronal damage induced by oxidative stress [32]. Moreover, it exerts beneficial effects on bone development in mice [33].

Fibroblast growth factor 21 (FGF21) is a brown adipokine that promotes non-shivering thermogenesis response in humans. In 2014, Lee et al. compared the effect of cold exposure on irisin and FGF21 secretion, demonstrating that both show similar capacities to induce fat browning [34]. Interestingly, transgenic mice with ectopic expression of *uncoupling protein 1 (UCP1)* in skeletal muscle showed increased secretion of FGF21 from skeletal muscle, leading to increased browning of WAT [35]. Circulating FGF21 levels are elevated in metabolic diseases such as obesity, insulin resistance, and T2D mellitus [8,36,37,38]. In skeletal muscle, FGF21 is an insulin-regulated myokine, but it is released also in response to exercise, as well as mitochondrial dysfunction and endoplasmic reticulum (ER) stress. Indeed, FGF21 has been proposed as a biomarker of mitochondrial dysfunction in skeletal muscle [39]. FGF21 is produced and released into the circulation also by other organs, such as the liver, WAT, and heart [40]. It has been recently demonstrated that mice with specific deletion of *FGF21* in skeletal muscle are protected against starvation-induced muscle atrophy and weakness, indicating that skeletal muscle represents a target of muscle-derived FGF21. On the other hand, in vivo overexpression of *FGF21* in skeletal muscle induces autophagosome formation and muscle loss, supporting a role for FGF21 in skeletal muscle remodelling [40]. The Growth Differentiation Factor 15 (GDF-15) is a biomarker of cellular stress and it has been identified as an exercise-regulated myokine released upon muscle contraction. Regulation of GDF-15 production by exercise has been investigated by a recent preclinical study showing high levels of GDF-15, in the conditioned media from exercised human myotubes, activating lipolysis in human adipocytes in vitro [41,42,43], to support a role for this myokine in regulating adipose tissue function.

Myonectin, alternatively known as C1q tumor necrosis factor α-related protein isoform 15 (CTRP15), is upregulated in skeletal muscle during exercise as well, and it affects adipose tissue and liver functions. Indeed, myonectin-knockout mice fed a high-fat diet showed a significant increase in adiposity due to greater lipid storage, resulting in hyperthorpic adipocytes. In parallel, *myonectin* deletion also modified lipid accumulation in the liver, in terms of a significant decrease in triglycerides and cholesterol content, thus resulting in reduced liver steatosis. Such observations revealed that the absence of myonectin leads to different lipid distribution between adipose tissue depots and the liver [44].

Skeletal muscle produces and releases interleukin-6 (IL-6) after prolonged exercise. More specifically, IL-6 is secreted by both adult skeletal muscle and satellite cells in response to muscle stress, thus contributing to modulate the classical myogenic process as well as to muscle regeneration [45]. Interestingly, skeletal muscle release of IL-6 is regulated by substrate availability (glycogen) during exercise, thus indicating that it acts as an energy sensor [46]. It has been shown that in humans, IL-6 contributes to hepatic glucose production and enhances fat oxidation and lipolysis in skeletal muscle during exercise [47,48,49]. Interestingly, despite the well described pro-inflammatory effects of systemic IL-6 in obesity and metabolic diseases, clinical studies show that IL-6 secreted by contracting skeletal muscle exerts anti-inflammatory effects and promotes favourable metabolic responses, also increasing insulin sensitivity [50]. IL-6 plays a pivotal role in the regulation of both adipose tissue and skeletal muscle metabolic processes, also contributing to the cross-talk between these tissues. In particular, IL-6 released from skeletal muscle communicates with adipose tissue to induce lipolysis during fasting or exercise [51]. Moreover, IL-6 derived from skeletal muscle has been shown to suppress macrophage infiltration in adipose tissue [52]. On the other hand, in adipose tissue, IL-6 signaling pathway activation promotes macrophage infiltration, leading to a chronic state of low-grade inflammation, as well as to obesity-related insulin resistance [51,52].

It is important to highlight that despite the large number of identified myokines, to date, only few of them have been fully characterized. Therefore, it is important to investigate their role in skeletal muscles under physiological and pathophysiological conditions. Of note, irisin and FGF21 have attracted increasing attention in recent years due to their potential beneficial roles in metabolic homeostasis, but more studies are necessary to clarify their role [28,53]. Therefore, taking advantage of in vitro and in vivo models is mandatory to investigate the molecular mechanisms driving skeletal muscle metabolic dysfunctions. Appropriate preclinical models are available (see below) to study the impact of altered skeletal muscle function and myogenesis on myokine production.

## 3. Myogenesis Regulation and Skeletal Muscle Quality Maintenance

Skeletal muscle is composed by different tissues including myofibers, blood vessels, nerve fibers, and connective tissue. Each skeletal muscle shows three layers that surround it, which are composed by connective tissue, whose function is to protect as well as to provide structure to the muscle and to arrange muscle fibers. Such structural and function complexity is essential to accomplish the task of generating contraction, force, and movement. In vertebrates, abnormal nutritional environments have been demonstrated to inhibit myoblast differentiation, decreasing myofibers’ number [54,55,56]. Indeed, skeletal muscle originates from cells found in the mesoderm, and myogenic commitment, during development, is regulated by signaling factors released by the surrounding tissue [57]. It has been shown that maternal obesity enhances the expression of adipogenic markers in fetal skeletal muscle, also promoting systemic inflammatory response [58]. Accordingly, another in vivo study demonstrated that low-grade inflammation, which occurs in obesity, downregulates myogenesis and results in reduced fetal muscle fibers’ size [55].

In physiological conditions, myogenesis is characterized by an initial phase of precursor cells’ proliferation, followed by the expression of muscle-specific markers, and finally, the fusion of differentiating myoblasts into mature myotubes. Indeed, plenty of signaling molecules drive myogenesis during embryonic development and in postnatal life. Such signals are mediated by cell surface receptors, which in turn trigger intracellular pathways culminating in the activation of transcription and chromatin-remodeling factors. More specifically, in the developing embryo, the paraxial mesoderm forms blocks of cells called somites, which give rise to dermomyotome cells, expressing paired box transcription factors (Pax3/Pax7) and a family of transcription factors known as myogenic regulatory factors (MRFs), which assign myogenic identity to muscle progenitors [59]. The primary myotome originating from dermomyotome disintegration contains committed muscle cells expressing MyoD and Myf5, which are basic helix–loop–helix (bHLH) transcription factors, downstream of Pax3 and Pax7, and are considered markers of terminal specification to the muscle lineage [60,61,62]. Notably, progenitors forming primary myotome include satellite cells, residing in the quiescent state within mature muscles and undergoing myogenesis when muscle fibers are damaged, in order to repair the tissue and re-establish homeostasis [63,64].

In summary, during development, MRFs regulate the progression of skeletal muscle lineage with a specific hierarchy: Pax3/Pax7 are master regulators of early lineage specification, while Myf5 and MyoD are responsible for myogenic commitment. Subsequently, two more terminal MRFs are required for the myocytes’ fusion and myotube generation: myogenin (Myog) and MRF4 (also known as Myf6). In skeletal muscle, fiber size depends on balancing cell number and cell size to generate multinucleated muscle fibers, each one containing the appropriate number of nuclei. Such a delicate task is carried out by Myog, which regulates myofiber maturation and size, acting later than MyoD, Myf5, and MRF4 [65,66]. In vivo studies showed that among MRFs, Myog is necessary for embryonic muscle differentiation. Indeed, Myog-null mice die before birth or early in postnatal life due to defective muscle differentiation [67,68]. Interestingly, Knapp et al. abolished Myog expression 3 days after birth in mice, i.e., after embryonic skeletal muscle growth, and observed normal skeletal muscle development but reduced body size, thus indicating that Myog is important for skeletal muscle growth in postnatal life [69]. Although each MRF gene plays a specific role during myogenic development, a functional redundancy between Myf5 and MyoD has been described. Indeed, a null mutation of *MyoD* into the mouse germline resulted in an upregulation of Myf5 and normal muscle development [70], suggesting that if MyoD is inactive, Myf5 is able to compensate for it, carrying out the normal myogenic program. Nevertheless, satellite cells lacking both *Myf5* and *MyoD* fail to regenerate a healthy muscle, confirming that these two factors are crucial for skeletal muscle differentiation [71]. Notably, MyoD has been suggested as a potential negative regulator of brown adipogenesis due to the observation that *MyoD* deletion in murine myoblasts promotes brown adipogenic transdifferentiation [72]. Myogenic bHLH proteins form heterodimers with ubiquitous basic helix–loop–helix proteins known as E proteins, and they activate several other downstream muscle factors binding E boxes in regulatory regions of muscle target genes [73]. In addition, MRFs associate with members of the myocyte enhancer factor 2 (MEF2) family, which show binding sites in the promoters and enhancers of several skeletal muscle specific genes [74]. Although MEF2 alone does not show myogenic activity, it is essential for differentiation due to its property to potentiate bHLH activity [75,76]. Indeed, in *Drosophila*, a loss of function mutation of the *Mef2* gene only inhibits skeletal muscle differentiation [77]. Myogenic transcription is also controlled by different co-activators and co-repressors, which cooperate with MRFs to drive muscle gene expression. More precisely, histone acetyltransferases (HATs) and histone deacetylases (HDACs) both interact with MyoD, showing opposite effects. HAT activity is increased during myogenesis, and histone acetylation promotes MyoD binding to specific promoters [78]. On the other hand, HDACs negatively regulate myogenic differentiation through a direct interaction with MEF2 and MyoD [79].

Once myogenesis is completed, the contractile unit of skeletal muscle is represented by the sarcomere, which is composed of actin, myosin, and associated proteins such as troponin and tropomyosin. Skeletal muscle myosins are classified as class II, and they consist of two heavy chains (MyHCs), two essential light chains (MLCs), and two regulatory MLCs each. Most myosins are encoded by genes expressed in the developing skeletal muscle and are known as embryonic and neonatal myosins [80]. Notably, MyHC protein expression determines skeletal muscle phenotype. Indeed, four predominant *MyHC* genes are expressed in adult skeletal muscle (type I, IIa, IId/x, and IIb), which are associated with fiber types with corresponding names, showing different contractile speed and aerobic or anaerobic properties [81]. As discussed by Pette et al., MyHCI fibers (slow twitch) predominantly rely on oxidative metabolism and use lipids as fuel, whereas MyHCIIb fibers (fast-twitch) are mainly characterized by glycolytic metabolism and use glucose as an energy source. MyHCIIa and MyHCIIx show metabolic properties which are intermediate between type I and IIb fibers [82]. Notably, skeletal muscle fibers are capable of changing their phenotype, undergoing transitions from fast-to-slow and slow-to-fast in response to increased or decreased neuromuscular activity, mechanical loading or unloading, altered hormonal profiles, and aging [82]. Mammals have skeletal muscle groups with variable composition in fiber types, leading to specific functional properties [81]. Interestingly, the existence of different fiber types represents a form of skeletal muscle adaptation to whole body metabolism. In the presence of hyperglycemia, slow-oxidative muscle fibers are more efficient in removing glucose from blood compared to fast-glycolytic fibers. Indeed, an altered fiber types distribution with the prevalence of fast-glycolytic fibers is often associated with obesity, potentially contributing to the development of insulin resistance [83].

Mice carrying mutations in different *MyHC* genes show different phenotypes. For instance, *MyHC-IId* null mice exhibit chronic limb weakness, whereas mice null for the *MyHC-IIb* and *IId* genes are viable and fertile, but their weight is lower compared with wild-type controls of the same age [84]. Interestingly, Nielsen et al. demonstrated that resistance exercise induces an increase IL-15 mRNA levels in muscle groups dominated with a higher proportion of type 2 fibers in healthy subjects, although plasma-circulating levels were not increased [85]. Such an observation suggests that muscle fiber type composition could local affect myokine production in skeletal muscle, leading to potential different contributions to local and systemic metabolic regulation.

## 4. Skeletal Muscle Cell Cultures

Due to the complex structural arrangement of skeletal muscle, it is difficult to identify the specific contribution of muscle cells to muscle physiology as well as study intramyocellular processes in vivo. For this reason, primary human cells represent the best-established model so far. Human myogenic cell cultures can be obtained by skeletal muscle biopsies from adult donors and can undergo differentiation to form mature myotubes [86]. As already mentioned, they represent a valuable model to study many aspects of muscular function and disease, including insulin resistance. In addition, induced Pluripotent Stem Cells (iPSCs) reprogrammed into an embryonic-like pluripotent state allow the establishment of different cell type cultures, including myoblasts [87]. In particular, IPSCs display unlimited expansion potential, together with a marked ability to be differentiated in vitro, representing a unique opportunity to study several dysfunctions, including impaired insulin signaling, altered glucose tolerance, and decreased mitochondrial oxidation [88].

Rat L6 and mouse C2C12 cells, together with primary myogenic cells, represent the most commonly used cellular models to study skeletal muscle in vitro. Established myoblast cell lines, such as C2C12 cells, are skeletal myoblasts derived from the thigh muscle of mice and genetically modified to proliferate indefinitely and differentiate into myotubes cultures. Indeed, these cells are able to differentiate into myofibers expressing contractile proteins [89]. C2C12 myoblasts can be grown in Dulbecco’s modified Eagle’s medium (DMEM) containing glucose, fetal bovine serum (FBS), and antibiotics. At confluence, cell differentiation can be induced by switching to a low FBS or Horse Serum (HS) culture. Similarly, L6 cells have been isolated by Yaffe from cultures of the thigh muscle of newborn rats, and they are able to generate multinucleated myotubes [90].

There is evidence that such cell lines may be considered an appropriate model to investigate skeletal muscle metabolism as well as altered molecular pathways that promote muscle dysfunctions. Unfortunately, comparative studies of these cellular models are scarce. However, cell culture studies highlighted that similar stimuli are able to differentially modulate the metabolic response in distinct skeletal muscle cells models [91,92,93]. For instance, Mahfouz et al. demonstrated that sphingolipids treatment promotes insulin resistance in vitro by activating different pathways in C2C12 and L6 myotubes, probably depending on cell membrane structure or composition [92]. Similarly, glucocorticoids administration induces different muscle wasting responses in cultured L6 and C2C12 myotubes [94]. Given the well-established role of skeletal muscle mitochondria metabolism in insulin resistance as well as in T2D development, a recent study investigated mitochondrial respiration in L6 and C2C12 myoblasts and myotubes. Interestingly, L6 cells exhibited attenuated respiration rates compared with C2C12 myoblasts which, on the other hand, showed greater capacity for lipid-supported respiration [93].

These observations should be taken into account in order to choose the most appropriate model to investigate specific metabolic aspects.

## 5. Cellular Models to Investigate Insulin Resistance in Skeletal Muscle

Skeletal muscle insulin resistance represents a main driver of T2D and metabolic syndrome [95]. Myofibers represent the predominant site of insulin-mediated glucose uptake in the postprandial state in humans, and increased levels of circulating free fatty acids (FFAs) and inflammatory cytokines directly affect the insulin signaling network [96]. In this context, muscle bioenergetics failure significantly contributes to systemic metabolic dysfunctions.

In the past two decades, myoblast cell lines have been recognized as valuable experimental tools in several conditions including obesity, diabetes, and insulin resistance [97]. Both C2C12 and L6 myoblasts are able to undergo differentiation into elongated myotubes, expressing the insulin responsive glucose transporter 4 (GLUT-4) protein, which promotes insulin-stimulated glucose uptake [98,99]. In physiological conditions, high-glucose circulating levels stimulate pancreatic β-cells to secrete insulin, which drives, together with muscle contraction, glucose uptake into target cells, including myofibers [100]. Such a fine molecular mechanism is dependent on insulin binding to its receptor (IR), which in turn activates tyrosine phosphorylation cascade, eliciting GLUT-4 protein increase and translocation from the cytoplasm to the cell membrane [101,102]. In the presence of insulin resistance, skeletal muscle insulin sensitivity is reduced due to impaired IR function and GLUT-4 translocation [103]. The development of in vitro models of skeletal muscle insulin resistance provides useful tools to investigate the mechanisms underlying metabolic dysfunction. Different protocols have been proposed to mimic insulin resistance in vitro. Importantly, insulin resistance can be developed in both appropriate treatments of cell cultures during myogenesis or in terminally differentiated myotubes in vitro. It has been shown that C2C12 myoblast cultures’ exposure to high glucose (15 mmol/L) combined with high insulin concentration (50 nm/L) impairs the insulin-like growth factor-1 (IGF-1) signaling pathway in mature myotubes [104]. Interestingly, the administration of high insulin concentration is able to induce C2C12 myoblast differentiation only under high glucose conditions [105]. In the absence of insulin, high glucose levels are toxic due to ROS production, and they induce protein degradation in cell cultures [106]. As already mentioned, insulin is essential in mediating glucose uptake, but chronic exposure to high insulin concentrations impairs myoblasts metabolism. A preclinical study described the effects exerted by 72 h of insulin treatment in C2C12 myoblasts during differentiation or in mature myotubes. Insulin-stimulated glucose uptake was altered in myoblasts exposed to chronic insulin during differentiation, as revealed by attenuated Akt phosphorylation, indicating an impaired signaling upstream of Akt. Of note, chronic insulin exposure did not exert the same effect in mature C2C12 myotubes [107]. These findings indicate long-term incubation with insulin during differentiation as a valuable method to induce insulin resistance in C2C12 myoblasts. Similar effects have been observed in L6 cell cultures under high glucose/high insulin conditions for 24 h, where a significant defect in insulin-mediated glucose uptake, impairment in IR and IRS-1 phosphorylation, as well as Akt phosphorylation were observed in mature myotubes [108].

**Figure 2:**
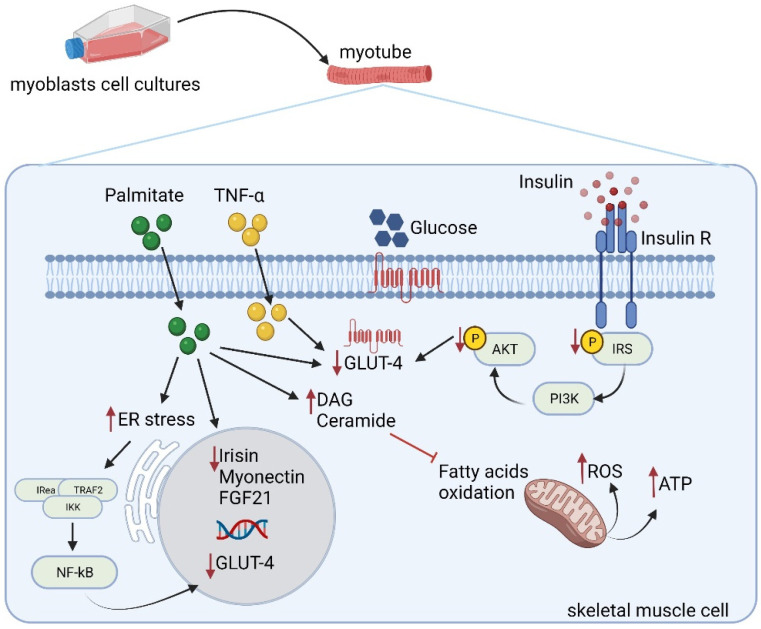
Schematic representation of insulin resistance induction in skeletal muscle cells. In the presence of insulin resistance, skeletal muscle insulin sensitivity is reduced due to impaired IR function and GLUT-4 translocation. Moreover, chronic exposure to high insulin concentrations impairs myoblasts metabolism. Palmitate is known to induce insulin resistance by impairing glucose uptake and reducing GLUT4 expression in skeletal muscle. Indeed, palmitate induces a significant reduction of insulin-stimulated Akt phosphorylation as well as GLUT-4 mRNA expression. In addition, palmitate promotes myotubes loss and impaired expression of irisin, myonectin, and FGF-21. Moreover, acute palmitate treatment promotes ER stress; disturbed ER homeostasis activates an adaptive process called unfolded protein response, leading to the formation of the IREa–TRAF2–IKK complex, which activates NFkB to translocate into the nucleus, downregulating GLUT-4 gene expression. Similarly, TNF-α has been reported to induce insulin resistance in skeletal muscle cells. Indeed, differentiated primary muscle cells cultured in the presence of TNF-α showed GLUT-4 translocation impairment. Red arrows indicate positive or negative modulation of downstream targets. Red line indicates an inhibitory effect mediated by DAG and ceramide on fatty acids oxidation. Abbreviations: ER, endoplasmatic reticulum; FGF21, fibroblast growth factor 21; ROS, reactive oxygen species; TNF-α, tumor α necrosis factor; NFKB, nuclear factor kappa B; TRAF2, TNF receptor-associated factors; IKK, I kappa B kinase; DAG, diacylglycerols; IRea, serine/threonine-protein kinase/endoribonuclease.

Increased plasma FFA levels are observed in obese/diabetic subjects, and several studies provided evidence that FFAs play a role in insulin resistance development [109,110]. In vitro insulin resistance can be induced by saturated fatty acids administration (Figure 2). Indeed, treatment of C2C12 myotubes with 0.75 mM palmitate and 2% bovine serum albumin for 24 h induced a significant reduction of insulin-stimulated Akt phosphorylation as well as *GLUT-4* mRNA expression, leading to an insulin-resistant phenotype in vitro [111]. In addition, palmitate promoted myotubes loss in C2C12 myotubes and impaired the expression of myokine, such as irisin, myonectin, and FGF-21, whose role in metabolic homeostasis has been already discussed above [111]. Similarly, both chronic and acute palmitate exposure significantly reduced *GLUT4* mRNA and protein expression in L6 myotubes, leading to an inflammatory state and insulin resistance, [112,113,114] with potential involvement of endoplasmic reticulum (ER) stress. In fact, acute palmitate treatment promotes ER stress, resulting in unfolded and misfolded proteins. Disturbed ER homeostasis activates an adaptive process called unfolded protein response, leading to the formation of the IREa–TRAF2–IKK complex, which in turn activates NFkB to translocate into the nucleus, downregulating *GLUT-4* gene expression [114]. Notably, in muscle cells, palmitic acid treatment also impairs mitochondrial function in association with decreased insulin response. Both C2C12 and primary rat skeletal muscle cells cultures, upon FFA exposure, show decreased insulin-induced glycogen synthesis, glucose oxidation, and lactate production with a parallel impairment in mitochondrial function and reduced ATP generation in response to mitochondrial substrate pyruvate [115] (Figure 2). Similarly, TNF-α has been reported to induce insulin resistance in skeletal muscle cells. Indeed, differentiated primary muscle cells isolated from rats and cultured for 24 h in the presence of TNF-α showed impairment of both insulin-stimulated glucose uptake and GLUT-4 translocation [116]. Notably, chronic oversupply of metabolic fuel through glucose/palmitate administration was able to induce insulin resistance in L6 myotubes, promoting pro-inflammatory signaling with a parallel reduction in mitochondrial respiration capacity and key mitochondrial proteins expression as well as mitophagy increase [117] (Figure 2). Such observations indicate that all these stimuli not only induce an impairment in insulin signaling and glucose uptake but directly impact several signaling pathways regulating mitochondrial fuel oxidation and respiration, negatively affecting skeletal muscle energy homeostasis [118,119].

As mentioned above, iPSCs have been recently extracted from patients with T2D and differentiated into myoblasts and exhibit a clear phenotype of insulin resistance in vitro. These cells are characterised by altered insulin signaling, decreased insulin-stimulated glucose uptake, and reduced mitochondrial oxidation [88]. iPSCs, isolated from T2D patients’ biopsies, can be induced to differentiate into skeletal muscle myoblasts (iMyos) in a two-step protocol, as previously described by Caron et al. [120]. Notably, iMyos displayed defective insulin response in vitro with no requirement to apply specific stimuli to trigger insulin signaling impairment. This brilliant model is able to reproduce skeletal muscle insulin resistance, occurring in patients with T2D, in a culture dish, opening novel unexpected perspectives to explore the mechanisms of insulin resistance in peripheral tissues.

## 6. In Vivo Models to Investigate Insulin Resistance in Skeletal Muscle

Due to skeletal muscle structural and functional complexity, a deeper comprehension of its contribution to glucose homeostasis and local and systemic metabolic dysfunction development requires animal models. To date, several models are available to investigate insulin resistance in vivo. Rodents (mice and rats) represent the most common models employed in metabolic studies. Interestingly, the generation of mice defective for the insulin receptor (IR) showed that IR is essential for postnatal fuel homeostasis but not for embryonic muscle growth and metabolic control. Indeed, IR KO mice die after birth due to β-cell failure followed by diabetic ketoacidosis [121]. On the other hand, muscle-specific IR KO mice develop metabolic syndrome features, including high triglycerides and free fatty acids circulating levels, although they do not show altered glucose tolerance, thus indicating that other organs, such as the liver and adipose tissue, are able to compensate skeletal muscle insulin resistance to avoid hyperglycaemia and hyperinsulinemia, leading to T2D [122].

In humans, obesity and T2D are closely linked. Similarly, many animal models of insulin resistance are characterized by the occurrence of obesity. The most commonly used models include Lep^ob/ob^ and Lep^db/db^ mice, which are deficient in *leptin* and *leptin receptor*, respectively, and thus are defective in leptin signaling. Both these models are hyperphagic and show a typical phenotype characterized by weight gain, hyperinsulinemia, hyperlipidemia, dysfunctional thermoregulation and reduced physical activity. Similarly, Zucker fatty rats and Zucker diabetic fatty rats are deficient in leptin receptor and show hyperphagia, obesity, hyperinsulinemia, hyperlipidemia, and hypertension [123]. Notably, muscle atrophy in type 2 diabetic rodent models is less consistent than in humans. Several preclinical models, including mice with diet-induced obesity or IR KO mice, exhibit a mild decrease in muscle size compared to Lep^ob/ob^ mice, which show a severely reduced muscle size [124,125,126]. In particular, leptin gene deletion induces altered fiber proportions in obese mice muscles, displaying a prevalence of slow-type myofibers. Changes in myofiber types are responsible for a significant prolongation of contraction and relaxation times in this model. Obesity-associated physical inactivity plays a crucial role in generating the effects on myofiber type composition observed in Lep^ob/ob^ mice. [127]. Similarly, Lep^db/db^ mice display muscle atrophy, as well as slow contraction and relaxation [128]. Skeletal muscles in these mice show an increase in protein degradation due to both dowregulated PI3K/Akt signaling and increased proteolytic activity of the ubiquitin–proteasome proteolytic pathway (UPP) [129]. In addition to reduced contraction induced by atrophy, the slower relaxion rate observed in this model is probably due to a decreased content in the muscle sarcoplasmic reticulum Ca^2+^ adenosine triphosphatase protein (SERCA) pump [128]. Muscle atrophy and altered activation of insulin-dependent signaling pathways (PI3K/Akt) may contribute to impaired glucose metabolism in Lep^ob/ob^ and Lep^db/db^ mice. IR substrates (IRS1 and IRS2) belong to the IRS protein family and play a pivotal role in insulin/IGF-1-signaling, regulating Akt/mTOR and FoxO pathways [130]. Interestingly, double knockout mice with combined deficiency of IRS1 and IRS2 in muscle display reduced skeletal muscle growth due to dysregulated glucose metabolism, which may also be a consequence of reduced muscle mass and insulin sensitivity.

Of note, a considerable proportion of normal-weight subjects shows insulin resistance features [131], suggesting that obesity is not required to trigger a condition of insulin resistance. Thus, the availability of non-obese mice with altered insulin signaling provides models suitable for investigating pathophysiological mechanisms underlying the insulin-resistant lean phenotype. Transgenic mice with muscle-specific overexpression of *lipoprotein lipase (LPL)*, a well-known rate-controlling enzyme involved in triglyceride hydrolysis [132], represent a valuable model to selectively increase fatty acids delivery to skeletal muscle and investigate insulin signaling in this tissue. Importantly, mice overexpressing *LPL* in skeletal muscle show impaired glucose tolerance with a 50% decrease in insulin-stimulated glucose uptake in gastrocnemius, confirming the key role of skeletal muscle in the regulation of systemic glucose metabolism. Skeletal muscle insulin-stimulated glycolysis and glycogen synthesis were also significantly decreased, which is in accordance with the impaired insulin signalling of this model. Notably, despite a decrease in insulin-stimulated PI3-kinase activation, insulin receptor function was intact in muscle-specific *LPL* overexpressing mice, indicating that insulin resistance is due to a mechanism occurring downstream the IR [133].

Physical activity is a potent mediator of insulin sensitivity [134]. Prolonged physical inactivity correlates with obesity, insulin resistance, and T2D mellitus [135]. On the other hand, it is well established that regular physical activity reduces inflammation and improves insulin resistance [136]. Of note, several studies demonstrated that skeletal muscle short-term disuse reduces lean mass more severely in older subjects than in young subjects, independently from baseline muscle sizes [137,138,139]. These findings have been confirmed in rats after 42–48 h of immobilization, resulting in a decreased insulin-stimulated glucose uptake in both fast-twitch and slow-twitch red fibers in muscles of immobilized mice. In this study, reduced insulin sensitivity in skeletal muscle was independent of glucose transporter, insulin receptor abundance, or receptor kinase activity, and it was rescued by the combined action of insulin and physical exercise [140].

## 7. Conclusions

For several years, skeletal muscle has been an underestimated target in metabolic disease investigation, although its contribution to overall body mass and structural conformation, as well as its ability to regulate metabolism via interorgan cross-talk, have been demonstrated to significantly impact general health and well-being.

It is well established that decreased muscle contraction and impaired skeletal muscle metabolism in terms of altered insulin signaling and/or mitochondrial dysfunction play a major role in the development of metabolic diseases such as obesity, insulin resistance, and T2D, leading to cardiometabolic complications [141]. A detailed knowledge of the molecular pathways regulating muscle differentiation and metabolism is a critical requirement for developing strategies to treat the above-mentioned diseases. To date, employment of human as well as murine cellular models has provided a growing body of evidence that helped to elucidate the contribution of skeletal muscle to energy homeostasis. In particular, myoblasts cultures differentiated in vitro are fundamental tools for understanding the molecular mechanisms regulating myofiber differentiation, which, in turn, determine muscle ability to perform physiological contraction, secrete myokines, and regulate glucose tolerance in this tissue. In addition, cell cultures allow mimicking insulin resistance conditions in vitro thanks to several stimuli affecting different molecular pathways involved in insulin signaling.

On the other hand, studies with animal models are required to investigate the multiple metabolic connections between skeletal muscle and other tissues involved in glucose homeostasis, such as heart, fat, and liver. In summary, the use of proper preclinical models of skeletal muscle dysfunction is mandatory to explore the molecular pathways causing insulin resistance and to help the development of proper pharmacological, nutritional, and physical rehabilitation interventions in the context of the cardiometabolic syndrome.

## Abbreviations

T2D	Type 2 diabetes
MSTN	Myostatin
BAT	Brown adipose tissue
WAT	White adipose tissue
FST	Follistatin
AMPK	AMP-activated protein kinase
PGC1α	Peroxisome proliferator-activated receptor-gamma co-activator
FNDC5	Fibronectin type III domain-containing protein 5
FGF-21	Fibroblast growth factor 21
UCP-1	Uncoupling protein 1
GDF-15	Growth differentiation factor-15
IL-6	Interleukin-6
Pax3	Paired box 3
Pax7	Paired box 7
bHLH	Helix–loop–helix
MRF	Myogenic regulatory factors
MyoD	Myoblast determination protein 1
Myog	Myogenin
Myf5	Myogenic factor 5
MEF2	Myocyte enhancer factor 2
MyHC	Myosin heavy chain
GLUT-4	Glucose transporter 4
IR	Insulin receptor
IGF-1	Insulin-like growth factor-1
IRS-1	Insulin receptor substrate 1
IRS-1	Insulin receptor substrate 1
iPSCs	Induced pluripotent stem cells
LPL	Lipoprotein lipase
FFA	Free fatty acid
